# Overcoming Barriers to Agriculture Green Technology Diffusion through Stakeholders in China: A Social Network Analysis

**DOI:** 10.3390/ijerph17196976

**Published:** 2020-09-24

**Authors:** Wenke Wang, Jue Wang, Kebei Liu, Yenchun Jim Wu

**Affiliations:** 1 Business School, Sichuan Normal University, Chengdu 610101, China; wangwk@sicnu.edu.cn (W.W.); 2017190342@stu.sicnu.edu.cn (J.W.); 2018190920@stu.sicnu.edu.cn (K.L.); 2 Sichuan Provincial Key Laboratory of Sci-tech Finance and Mathematical Finance, Sichuan University, Chengdu 610064, China; 3 Graduate Institute of Global Business and Strategy, National Taiwan Normal University, Taipei 106, Taiwan; 4 College of Management, National Taipei University of Education, Taipei 106, Taiwan

**Keywords:** agriculture green technology diffusion, stakeholders, social network analysis, barriers

## Abstract

It is crucial to actively encourage the development of agriculture green technology, which has been regarded as one of the most effective solutions to the environmental degradation caused by agricultural activities. However, agriculture green technology diffusion is indeed a challenging task and still faces numerous barriers. The stakeholders who can potentially deal with these barriers, however, have been overlooked by previous studies. To address these issues, social network analysis was performed to identify critical stakeholders and barriers. Their interactions in agriculture green technology diffusion were analyzed based on the literature, a questionnaire survey and expert judgments. A two-mode network and two one-mode networks were used to analyze the relationships among the identified 12 barriers and 14 stakeholders who can influence these 12 barriers identified. The results show that agricultural research institutes, universities, agribusiness, agencies of township promotion, the government and farmers’ relatives are key stakeholders and that the limited market demand for green technology and the high cost of its diffusion are two main barriers. However, poor green technology operability and farmer families in distress are factors that are not as important as previously perceived. Finally, some recommendations and suggestions are provided to promote agriculture green technology diffusion in China.

## 1. Introduction

With the implementation of the rural revitalization strategy, China has experienced rapid development of agriculture and realized socialized etiquette and civility, effective governance and prosperous life. However, agriculture is the second-largest contributor to greenhouse gas emissions. To a large extent, its high-energy-consuming production technology has caused various environmental problems, which have greatly affected the development of agriculture [1]. Therefore, many scholars have conducted research related to this subject, and their results have shown that green technology can help reduce resource consumption and environmental pollution, while optimizing output [2]. Agricultural green technology aims to produce safe, pollution-free, high-quality, nutritious and green agricultural products. It is a group of technologies composed of multiple sub-technologies, including those related to pre-production (new variety of technology), mid-production (formula fertilization by soil testing, biological control technology of plant diseases and pests) and post-production (straw recycling technology). The diffusion of these technologies is a necessary condition for realizing food safety, and it provides the foundation and guarantee for the development of green agriculture [3]. Agriculture green technology can be adopted throughout the entire process of production, development and use of agricultural products. In recent years, developing countries have attached great importance to the development of agriculture green technology, which has promoted the creation of a green environment [4].

As a result of the numerous sustainable benefits of agriculture green technology, many governments and organizations have taken measures to promote its diffusion and adoption. However, it is still a challenging task. Based on the experience of diffusing green agricultural technology—such as green pest control, soil nutrient management (such as soil testing formula technology), conservation tillage and precision agriculture—it has been concluded that, even if a technology is useful, there are different factors that limit its effectiveness [5]. In research on the diffusion of agricultural technology, factors that inhibit its diffusion include uncertainty, information asymmetry, the number of adopters, land transfer and the credibility and authority of policymakers [6]. Lambrecht et al. analyzed the process of agricultural technology diffusion in eastern Congo, which showed that only 13% of farmers with an awareness of new technology adoption were willing to try it, and as many as 70% of farmers who tried a new technology were willing to continue using it [7]. To promote the diffusion and adoption of green technology, scholars have conducted many studies from different perspectives. These studies include (1) the impact of individual characteristics of farmers on the adoption of agriculture green technology [8]; (2) the impact of household resource endowment on the adoption of agriculture green technology [9]; (3) the impact of green agriculture promotion systems on the adoption of agriculture green technology [10]; and (4) the impact of economic risks on the adoption of agriculture green technology [11]. These studies have provided valuable information and ideas to guide developing countries in formulating policies that promote the diffusion and adoption of agriculture green technology.

In the process of promoting the diffusion of agriculture green technology, stakeholders play a vital role. Collaboration among stakeholders is an important step and integral in promoting the diffusion of agriculture green technology [4]. However, previous studies have overlooked stakeholder collaboration as a key to promoting agriculture green technology diffusion. The diffusion of agriculture green technology is increasingly influenced by diverse stakeholders due to factors such as farmers’ demand, public pressure, market and supply chain pressure and government policies. Only the effective interaction of multiple stakeholders can promote the diffusion of agriculture green technology and improve the overall environmental performance. The organization, development and implementation of agriculture green technology diffusion require stakeholders to have inner motivation to stimulate the development of innovative diffusion activities. Inner motivation is mainly derived from objectives, diffusion capabilities and the diffusion power of stakeholders [12]. However, driven by self-interest, stakeholders may invest resources in individual goals, rather than collective needs. If serious consequences occur, stakeholders tend to shirk responsibility and pass it on to others [13]. Therefore, problems related to the process of green technology diffusion are mainly caused by insufficient collaboration among stakeholders [14]. To build effective collaboration among stakeholders in the diffusion of agriculture green technology, it is necessary to understand the power of each stakeholder to remove barriers. Power reflects the abilities of stakeholders to overcome specific barriers. Stakeholders with a high degree of power should be primarily responsible for related barriers [12]. Otherwise, conflicts may arise between stakeholders due to a mismatch of power and responsibility. Stakeholders should actively assume relevant responsibilities according to their power and abilities, which is a prerequisite for effective stakeholder collaboration in agriculture green technology diffusion.

At present, a limited amount of research has been conducted on stakeholder collaboration in the diffusion of agriculture green technology and the removal of barriers to diffusion by stakeholders. Researchers and practitioners agree that the diffusion of agriculture green technology is more complex and challenging because agriculture is “traditionally very conservative” and changes slowly. Therefore, it is of vital importance to clearly identify the relationship between stakeholders and diffusion barriers and identify crucial factors that affect the diffusion. In order to fill the gap in this aspect, this paper first analyzes barriers to the diffusion of agriculture green technology from the perspective of stakeholders. Then, a comprehensive analysis of stakeholders and barriers is provided, and their impact on the diffusion of agriculture green technology is quantified through the social network analysis (SNA) method. Finally, the relationship between different stakeholders and barriers is explored, and key stakeholders and barriers, as well as their impact, are also identified. To the best of our knowledge, this paper fills gaps in previous studies on green agriculture, which have rarely involved the relationship between different stakeholders and barriers. In our research, in contrast to traditional social network analysis, the one-mode network analysis examines the internal relationship in the same group of entities, while the two-mode network analysis explores the relationship between two different groups. The results of these analyses reveal interlinked stakeholders through the barriers they can jointly address and interlinked barriers through the stakeholders who have the power to influence them. Furthermore, the analysis of barrier factors from the perspective of stakeholders provides a useful reference for future research. In the process of agriculture green technology diffusion, the relationship between barriers and stakeholders, as well as the network characteristics of stakeholder collaboration, can be understood through this network model, and the cooperative relationship between stakeholders who overcome these barriers is also determined. Innovative viewpoints in this research can guide stakeholders to make correct decisions. This research not only enriches the theory of agriculture green technology diffusion and adoption but also provides insights into how China can effectively promote it and thereby facilitate agriculture development.

The rest of the paper is structured as follows: Section 2 reviews barriers to the diffusion of agriculture green technology and the related stakeholders, and Section 3 introduces social network analysis and the research methods adopted. Section 4 reports the research results and related indicator analysis, and Section 5 presents a discussion of related research. The conclusions and limitations of this study and future research directions are described in Section 6.

## 2. Literature Review

### 2.1. Barriers to the Diffusion of Agriculture Green Technology

The diffusion of green technologies refers to the process in which the source of innovation disseminates innovative green technologies among individuals and diffuses them throughout all of society [15]. It is a process in which new agricultural technologies, inventions and results are widely adopted by farmers over a certain period of time and by several channels, after going through the stages of cognition, decision-making, participation and implementation and acknowledgment [4]. The diffusion of agriculture green technology not only drives the spreading efficiency of general technological innovation but also purposefully integrates human technological innovation activities into the natural ecological cycle. As a result, it will not only increase the income of farmers but will also reduce the damage to the environment caused by current agricultural technologies and protect natural resources to a certain extent [16]. However, agriculture green technologies are developing and diffusing slowly [17].

At present, excessive consumption of the Earth’s resources, a deteriorating environment and severe ecological damage have led to an increasing demand for agriculture green technology [2]. Although increasing importance has been attached to the development of green agriculture, and great efforts have been made to promote green technologies, problems still arise during the diffusion process and need to be resolved. Drawing on previous research, this paper systematically analyzes the content of the literature and determines corresponding barriers [18]. First, related journals and conference articles were searched in the databases of “Web of Science”, “Compendex Engineering Village”, ASCE library and CNKI, using keywords such as “barrier”, “green”, “agriculture green technology”, “agriculture technology”, “eco-friendly technology” and “ecological technology”. By reviewing the abstracts and conclusions, the articles that met relevant requirements were assessed to determine their relevance to this study. When the objectives were identified, a content analysis was performed based on the TOE (technology–organization–environment) framework, which has been widely applied in the technology acceptance field [19]. Taking the various barriers that affect agriculture green technology as the research object, we identified and analyzed barriers to the diffusion of green technologies in terms of technology, organization and the environment.

#### 2.1.1. Technological Barriers

The speed and effect of the diffusion of agriculture technologies are closely related to their nature. Currently, developing countries are putting more effort into innovating single agriculture technologies, while having relatively few supporting technical achievements and lacking effective technological support [10]. Therefore, it is difficult for them to adapt to the requirements of the development of green agriculture. This is also one of the important reasons that the diffusion of green technologies is difficult. As a result of the uncertainty and market risk of green technologies, many small and medium-sized enterprises are reluctant to make the investment in this field, which leads to insufficient investment in scientific research [20]. Insufficient technological innovation has led to a shortage in the supply of agriculture green technology.

During the diffusion of agriculture green technology, quite a few technology research institutes, limited by their own problems [21], do not have a comprehensive and in-depth understanding of the application of agriculture green technology, such as scarce scientific and technological resources, serious brain drain on green technologies and less tightly-bounded integration of industry, education and research. As a result, current technologies cannot meet market demand, and the diffusion of agriculture green technology faces difficulties. Furthermore, the level of informatization in green technologies also affects the spread of agriculture green technology [22]. In rural regions, geographic and information barriers make the channels through which farmers can obtain agriculture green technology relatively unidimensional. In addition, the poor applicability of present agriculture green technology and high diffusion costs have further impeded the spread of agriculture green technology.

#### 2.1.2. Organization Barriers

The diffusion of agriculture green technology involves the active organization and participation of different stakeholders. Decisions made by enterprises and governments are of great significance to the spread of agriculture green technology. Relevant policies under implementation lack substantive supporting policies that could effectively promote the diffusion of green technologies [23], which has led to incomplete systems of research and science and promotion. Moreover, they face problems such as the disconnection between technology and production, rigid mechanisms and insufficient guarantees, so they cannot realize the development of modernized green agriculture. The diffusion of agriculture green technology will be seriously hindered by inadequate government policies and measures and poor organization [24].

Demonstration, application and promotion by agriculture technology workers are needed to transform agriculture green technology into productivity. As the work to promote agriculture technologies is not fully implemented by the associated government departments, promotion personnel have not received complete training in promoting agriculture green technology or in providing the necessary education. As a result of the aging of knowledge and a low level of personnel quality, farmers could not receive adequate technical guidance and technological upgrades, which will have an influence on the diffusion of agriculture green technology [25]. Moreover, as the farmers themselves have a low level of literacy, they are not capable of recognizing and applying agriculture green technology. This will further restrict farmers from using emerging agriculture green technology and restrain the spread of agriculture green technology [26].

#### 2.1.3. Social and Market Barriers

Developing countries are still lagging behind in the market of agriculture green technology. On the one hand, farmers in these regions have a relatively low level of literacy [27]. They are conservative, show little interest in new technologies and new things, have a poor capability of acceptance and want to avoid all the associated risks. Therefore, they are skeptical of agriculture green technology, thus reducing their need in this regard [28].

The diffusion of agriculture green technology is also subject to the market environment. Great market risks and uncertainties in the innovation of agriculture green technology [29] make it hard to achieve a return on investment, thus making it difficult to obtain investment from financial institutions. Meanwhile, changes in farmers’ preferences and reference groups, such as farmer households and family members, as well as market factors, have also influenced the adoption and diffusion of agriculture green technology. Familiarity with the product and the brand effect are key factors that influence the diffusion of agriculture green technology [30].

### 2.2. Necessity of Research on Stakeholders

With the transition of stakeholder theory from the dyadic perspective to the network perspective, cooperation among stakeholders has become a new goal in the field of stakeholder management [31]. Stakeholder cooperation means that a group of self-governing stakeholders take actions and make decisions on related issues by using shared rules and standards [32]. Factors such as conflict of interest, different opinions and complicated relations will impede cooperation [33]. A good collaboration of interest and relationships among stakeholders is helpful for achieving full integration and optimization of resources so as to realize successful coordination among stakeholders.

Power is one of the most salient attributes of stakeholders [34]. Power means the ability of social actors to change others’ behaviors, so as to achieve their own objectives, regardless of opposition [35]. The power of stakeholders depends on a number of factors, such as the resources that they own [13] and their personal attributes and social status [36]. During their collaboration, stakeholders who have the most important resources will have more power [34]. Furthermore, the social network also highlights the strength of the structural positions of stakeholders [37], and the resources of stakeholders interact with their network positions. Therefore, it is necessary to understand the power of stakeholders and to determine their positions and relationships in the collaboration network, their attributes and the resources that they own.

In terms of research methods, the past decade has witnessed a number of studies that examined the factors of agriculture technology adoption using social network analysis. Wang et al. analyzed the efficiency of farmers’ agricultural technology adoption and the interaction effect between the social network and extension service [38]. In their research, interaction and trust had positive effects on farmers’ technology adoption efficiency, which was also reflected in our research. Ramirez discussed the influence of social networks on agricultural technology adoption; the study addressed the professional collaboration found in tenure relations and social and professional organizations, and the analysis showed that participation in organizations and kinship relationships are factors that influence agricultural technology adoption [39]. An interesting finding is that kinship relationships and township promotion agencies are also very important factors of stakeholders in our research. Kuang analyzed the reliability of interpersonal communication based on social networks and their information costs and found that the interpersonal communication of information established in social networks is most effective in the diffusion of agricultural technology [22], which is also a key stakeholder in our research.

Previous studies have shown that various barriers related to technologies, organizations, markets and social environments in the spread of agriculture green technology have inhibited the adoption and diffusion of agriculture green technology. At the same time, the diffusion of green technologies involves many stakeholders. Prior research and studies on the power and collaboration of stakeholders have suggested that the more powerful these stakeholders are, the more influence they have on the spread of green technologies. Effective collaboration among stakeholders will definitely have positive effects on the efficiency and performance of the diffusion of agriculture green technology. The existing research has barely explored how to effectively eliminate barriers to the diffusion of agriculture green technology from the perspective of stakeholders. Different resources provided by different stakeholders are needed to overcome these barriers. As a matter of fact, it is essential to study the influence of stakeholders on barriers to the diffusion of agriculture green technology, so as to improve the performance of this process.

## 3. Research Methods

This paper analyzes the complicated relationship between stakeholders and adoption barriers during the diffusion of agriculture green technology through social network analysis. Social network analysis is applied to examine the existence and strength of the relationship between a pair of stakeholders and adoption barriers. This theory emphasizes the “structure and mode” of the relationship and tries to identify the reasons behind adoption constraints and their corresponding influence [40].

### 3.1. Identification of the Stakeholders and Barriers

The establishment of social networks is based on nodes and links. This step mainly focuses on recognizing the network nodes, that is, finding the stakeholders of and barriers to the spread of agriculture green technology. First, the literature research method was used to define stakeholders and barriers [12]. Then, the Delphi method was used to identify more comprehensive and reasonable stakeholders and barriers through expert interviews using the data obtained in the first step [14]. The experts had to answer the following questions: (1) Does the literature research cover barriers or stakeholders that are not related to the diffusion of green technologies? (2) Can people understand the chosen barriers and stakeholders? (3) Are there any other barriers and stakeholders involved in the diffusion of green technologies?

In order to verify the validity and reliability of the chosen barriers and stakeholders, the experts interviewed all have rich research experience in this field and have unique insights into green technologies and the stakeholders involved. S* represents stakeholders and F# represents barriers, where * denotes the number of stakeholders and # is the number of barriers.

### 3.2. Determination of the Relationship between Stakeholders and Barriers

In order to analyze the influence of stakeholders on barriers to the spread of agriculture green technology, the snowball sampling technique, as well as questionnaires and expert seminars, were used to determine the relationship between stakeholders and barriers. Snowball sampling is particularly designed for cases that require professionals to give relevant opinions on issues in their field [41]. The questionnaires are mainly for researchers, managers and practitioners who have rich knowledge and experience in agriculture green technology [12]. The questionnaire was first distributed in November 2019. After modifying the questionnaire according to the opinions of respondents in a preliminary test, 297 respondents participated in the updated survey through a program named Questionnaire Star. The respondents include government administrators, company executives and workers at intermediary agencies, whose work is related to agriculture green technology, as well as research scholars and adopters of agriculture green technology. To ensure the reliability of the survey results, it was ensured that all respondents had more than three years of experience in studying, manufacturing and using agriculture green technology. In order to simplify the process, we used unified standards and determined the power of stakeholders on the barrier by applying an agreed-upon threshold value [42]. After the survey, a total of 11 expert representatives of agriculture green technology diffusion were invited to conduct workshops [14], including two developers, two market promotion representatives, two government administrators, two adopters and three academic researchers. All of them have more than 10 years of working experience and an in-depth understanding of agriculture green technology. A variety of methods were combined to expand the sample data. The process, including a survey with critical stakeholders and barriers and workshops with relevant experts, aims to minimize bias effects of dominant individuals and group pressure to conform. Therefore, the survey results and findings are of high accuracy from a scientific perspective. The result of this step is the definition of links in the stakeholder–barrier network.

An element in the stakeholder–barrier matrix refers to the condition in which a stakeholder handles barriers. It is composed of a group of stakeholders (X) and a group of barriers (Y). $X_{i}$ represents one of the 14 stakeholders, $Y_{j}$ denotes one of the 12 barriers, $a_{ij}$ indicates stakeholders and $X_{i}$ refers to whether one has the ability to solve barrier $Y_{j}$. The definitions are as below:

If $a_{ij}=1$, then stakeholder $X_{i}$ can resolve barrier $Y_{j}$;

If $a_{ij}=0$, then stakeholder $X_{i}$ cannot resolve barrier $Y_{j}$.

The stakeholder–barrier matrix was transformed into a stakeholder–stakeholder matrix and a barrier–barrier matrix. This can highlight the key stakeholders and barriers and evaluate the strength of the correlation on a quantitative basis.

### 3.3. Social Network Analysis

In contrast to traditional social network analysis, both the two-mode and one-mode network analysis were used to analyze the relationships between stakeholders and barriers, as well as the interrelationships between stakeholders or barriers, so as to build a good collaboration among stakeholders to address those barriers that are hard to solve.

#### 3.3.1. Two-Mode Social Network Analysis

In this study, the stakeholders can be regarded as the subject of the action of the network, and different kinds of barriers can be seen as the “organization” or the “case” affiliated to it. The two-mode social network analysis method analyzes the power of stakeholders in the social network to determine its impact and the means of overcoming obstacles. Using a two-mode social network analysis, Gan et al. provided a creative perspective on overcoming barriers to off-site construction by engaging stakeholders [12]. However, in our research, the closeness centrality of the two-mode network was further analyzed to explore stakeholders’ influence on the identified barriers.

1. Centrality analysis
  (1) Degree centrality
    Degree centrality measures the number of nodes directly linked to one node. It focuses on the degree of the power of nodes only. In the stakeholder–barrier network, this implies that stakeholders have the power to handle the number of barriers. As for barriers, the degree centrality is equal to the number of involved stakeholders [43]. The degree centrality can be represented by Equation (1). Therefore, the greater the degree centrality, the more associated the points, and the more important the position in the affiliated network.
    (1) $$C_{D}\left(K\right)=deg_{k}=\overset{N}{\underset{j}{\sum }}A_{kj}$$
    here, *K* represents the key node, *j* represents other nodes and *N* represents the total number of nodes. $A_{kj}$ represents an element in the matrix of stakeholders and barriers.
  (2) Closeness centrality
    The closeness centrality indicates the proximity of a network node to other nodes. The closeness centrality of a stakeholder refers to a function that represents the shortest network distance from the affected barrier to other stakeholders and barriers [44], which can be calculated by Equation (2). The greater the closeness centrality, the shorter the average distance to other nodes, and the more advantageous its position in the process of information transmission.
    (2) $$\;\;C_{C}^{NM}\left(n_{i}\right)={\left[1+\frac{\sum_{j=1}^{g+h}\underset{k}{\min}d\left(k,j\right)}{g+h+1}\right]}^{-1}$$
    here, $C_{C}^{NM}\left(n_{i}\right)$ represents the closeness centrality of stakeholders, *g* represents the number of stakeholders, *h* represents the number of barriers, *k* represents the barrier affected by a stakeholder, *j* represents some other stakeholder or barrier and $\underset{k}{\min}d\left(k,j\right)$ represents the shortest network distance between *k* and *j*.
    The closeness centrality of a barrier refers to a function that represents the shortest network distance from the affected stakeholder to other stakeholders and barriers [45], as shown in Equation (3).
    (3) $$C_{C}^{NM}\left(m_{k}\right)={\left[1+\frac{\sum_{i=1}^{g+h}\underset{j}{\min}d\left(i,j\right)}{g+h+1}\right]}^{-1}$$
    Generally speaking, the closeness centrality reflects the proximity of members of the network. The shorter the distance, the more likely it is to be at the center of the whole network. In Equation (3), $C_{C}^{NM}\left(m_{k}\right)$ represents the closeness centrality of a barrier, *g* represents the number of stakeholders, *h* represents the number of barriers, i represents the stakeholder affected by a barrier, *j* represents some other stakeholder or barrier and $\underset{j}{\min}d\left(i,j\right)$ represents the shortest network distance between *i* and *j*.
  (3) Betweenness centrality
    Betweenness centrality demonstrates the ability of a participant to play an intermediary role in the network [46], as is shown in Equation (4). It establishes the situation in which a node is located between two nodes and concentrates on the ability of a node to control the nodes at both ends. Only when each barrier affected by the stakeholder reaches a certain point will the stakeholder obtain betweenness centrality.
    (4) $$\frac{1}{2}\sum^{}n_{i},n_{j}\in m_{k}\frac{1}{X_{ij}^{N}}$$
    here, $n_{j}$ represents the other stakeholder of $n_{i}$ and the co-shared barrier $m_{k}$, and $X_{ij}^{N}$ represents the number of barriers shared by $n_{i}$ and $n_{j}$. In the same way, if a barrier only relates to one stakeholder, then the barrier will reach the middle “point” of ***g + h + 2***.
2. Core–periphery analysis

The core–periphery network has an ideal structure in which the rows and columns are divided into two categories. The block in the main diagonal represents the core, and the other one is the periphery [44]. In the stakeholder–barrier network, the “core” is composed of subdivisions of a series of stakeholders. These stakeholders are closely linked to each barrier in the subdivisions. The stakeholder at the core can be regarded as the key stakeholder of the network. The “periphery”, on the one hand, is composed of subdivisions that constitute a series of stakeholders that will not work together on the same barrier; on the other hand, it is composed of subdivisions of a series of barriers that are not correlated with each other [47]. This is because they are not connected by the same stakeholders. The core–periphery network structure can be expressed as Equations (5) and (6):

(5) $$\rho =\underset{i,j}{\sum }a_{ij}\sigma_{ij}$$

(6) $$\sigma_{ij}=\{\begin{array}{c} 1,if\;c_{i\;}=\;core\;or\;c_{j\;}=\;core\;\\ 0,other \end{array}$$

where $a_{ij}$ indicates whether there is a connection in the observed data, $c_{i}$ represents the category (core or periphery) of node *i*, $c_{j}$ denotes the category (core or periphery) of node *j* and $\sigma_{ij}$ indicates whether there is a connection in the real structure.

#### 3.3.2. One-Mode Social Network Analysis

During the process of agriculture green technology diffusion, the analysis of the stakeholder-barrier network can help identify the power of stakeholders over the barriers. Furthermore, it is a challenge for stakeholders to address multiple barriers as their resources are limited. By analyzing the stakeholder-stakeholder network and barrier-barrier network, the main stakeholders and barriers can be observed more clearly. The powerful stakeholders can be identified through the stakeholder-stakeholder network, thus the effective collaboration among them can be established to maximize their resources, so as to better address the barriers of agriculture green technology diffusion. While the barrier-barrier network can help stakeholders have a full understanding of the relationships between the barriers, and make the contributes to the decision making of agriculture green technology diffusion.

1. Network measures
  (1) Density analysis of the stakeholder/barrier network
    Density indicates the closeness of network members [48]. When the entire network has a greater density, it is more likely to influence the participants in terms of attitude and behavior. In return, the network is more complicated. The calculation of density is shown in Equation (7), with 0 ≤ density ≤ 1.
    (7) $$D\left(G\right)=\frac{K}{N\left(N-1\right)}$$
    here, D(G) represents the density of network *G*, *K* indicates the number of existing relationships and *N* represents the total node number of network *G*.
  (2) Cohesion analysis of the stakeholder/barrier network
    Cohesion indicates the relationship between two contacts of an action provider [48]. When cohesion is 0, there is no connection among the nodes; when cohesion is 1, there is a strong connection among the nodes, and any of them is capable of obtaining the same information and network benefits. Stronger cohesion means a closer relationship and a more complicated network. The calculation of cohesion is shown in Equation (8), with 0 ≤ cohesion ≤ 1.
    (8) $$C\left(G\right)=\frac{\sum^{}Adjm^{2}}{N\left(N-1\right)}$$
    here, C(G) represents the cohesion index, $Adjm^{2}$ represents the shortest distance between two nodes and *N* represents the total node number of the network.
2. In/out-degree analysis
  In/out-degree analysis reflects the strength of the influence of nodes. It divides the correlation strength between two nodes into outdegree and indegree. The outdegree of a node indicates the total number of nodes that it directly points to, while indegree indicates the total number of dots that other nodes point to [48]. The calculations are shown in Equations (9) and (10):
  (9) $$D_{D_{o}}\left(n_{i}\right)=\overset{n}{\underset{j}{\sum }}X_{ij}$$
  (10) $$D_{D_{i}}\left(n_{i}\right)=\overset{n}{\underset{j}{\sum }}X_{ji}$$
  where $D_{Do}\left(n_{i}\right)$ represents the outdegree, $X_{ij}$ represents the correlation between node *i* and node *j*, $D_{Di}\left(n_{i}\right)$ represents the indegree, and $X_{ji}$ represents the correlation between node *j* and node *i*. If there is a correlation, then the value is 1; if there is no correlation, then the value is 0.
3. Structural Hole analysis of the stakeholder/barrier network
  The structural hole indicates a non-redundant connection among actors. It offers opportunities for holders to obtain an “information benefit” and “control benefit”, so as to make them more competitive than members in another position of the network [48]. The parameters, range and formula are described below.
  (1) Effective size
    The effective size of an actor equals the non-redundant factors of the network in which the actor’s individual network deducts the redundancy of the network. It can measure the overall strength of the influence and the importance of nodes and indicates the control that a node has over the relationship between other nodes. The more effective the size of a node, the freer its action. The calculation of effective size is shown in Equation (11), with effective size > 0.
    (11) $$ES_{i}=s_{i}-\frac{2t}{s_{i}}$$
    here, *t* represents the number of correlations in the network, and $s_{i}$ represents the size of node *i*.
  (2) Efficiency
    Efficiency can be used to describe the influence of a node on other related nodes in the network. The efficiency of a node is equal to the ratio of its effective size to its actual size. The higher the efficiency of the node, the greater its influence in the control process. The efficiency of node *i* is calculated by Equation (12), with 0 ≤ efficiency ≤ 1.
    (12) $$EF_{i}=\frac{ES_{i}}{n}$$
    here, *n* represents the number of nodes and $ES_{i}$ represents the effective size.
  (3) Aggregate constraint
    Aggregate constraint indicates the ability of a node to use a structural hole. When a node of a single network is directly or indirectly connected to another one, it causes an aggregate constraint. The more aggregate constraint that a node has, the greater constraint that it has over the node, with 0 ≤ aggregate constraint ≤ 1. The aggregate constraint can be calculated by Equation (13):
    (13) $$c_{ij}=\left(p_{ij}+\sum^{}p_{iq}p_{qj}\right),\;i\neq q\neq j.$$
    In this formula, node *i* and node *j* both adjoin node *q*, $p_{ij}$ represents the weight ratio of node *j* among all the neighboring points of node *i*, and $p_{iq}$ and $p_{qj}$ represent the weight ratio of node *q* among all neighboring points of node *i* and node *j*, respectively.

## 4. Results

### 4.1. Establishment of the Relation Network

In this study, we performed a literature review and expert interviews to screen out stakeholders and barrier factors for agriculture green technology diffusion after two rounds of the Delphi survey. We invited 20 experts to attend the discussion group, including eight scholars who excel in green agriculture and 12 experts in green production and management of agriculture and agriculture technology. In round I, the background and purpose of this research was sent to respondents by e-mail. The respondents were instructed to screen out the stakeholders and barrier factors of agriculture green technology diffusion that had been collected and to list their reasons. In round II, all the data and information collected in round I were analyzed, and the results were provided to these experts to allow them to re-evaluate and judge the influencing factors. A total of 15 barriers and 20 stakeholders were determined preliminarily, but after documentary review and expert selection, a total of 12 barriers and 14 stakeholders remained, which are listed in Table 1.

**Figure 1:**
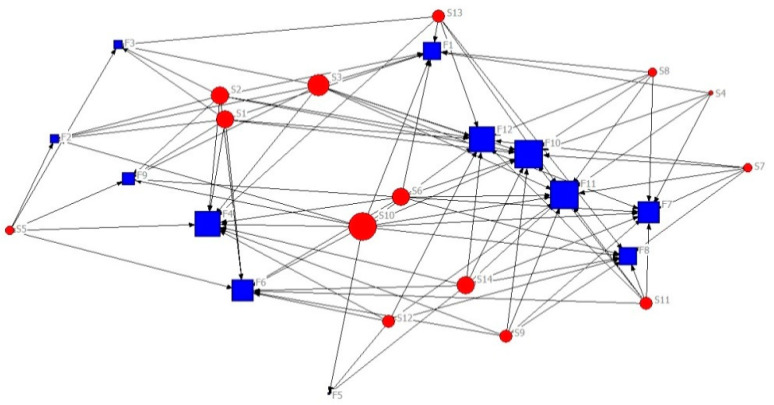
Visualization of the stakeholder–barrier network.

From the results of questionnaires and symposiums, the stakeholder–barrier network of agriculture green technology diffusion can be established by analyzing a relationships matrix between stakeholders and barriers and can be visualized through software [44], which is shown in Figure 1. The red circular nodes and blue rectangular nodes represent stakeholders and barriers, respectively, while the size of the nodes reflects the centrality. The key stakeholders and barriers are in the center of the network, so they have more links than other nodes. In a complicated system, a high cost of green technology diffusion and limited market demand for green technology are two major barriers that must be addressed by more stakeholders compared with other barriers. The main stakeholders that can influence barriers include agricultural research institutes, universities, agribusiness, agencies of township promotion, the government and farmers’ relatives.

### 4.2. Stakeholder–Barrier Network Analysis

#### 4.2.1. Centrality Analysis of Stakeholder–Barrier Network

Table 2 lists the centralities of the 14 stakeholders and 12 barriers that remained after analyzing the centrality of the stakeholder–barrier two-mode network. The results include degree centrality, betweenness centrality and closeness centrality

As shown in Table 2, the top three stakeholders in terms of degree centrality, betweenness centrality and closeness centrality are S10 (government), S3 (agribusiness) and S14 (farmers’ relatives), indicating that these stakeholders secure an important position in the network. The top one is S10 (government), indicating that this kind of stakeholder can solve many barriers with a variety of resources, and is closely related to other objects. It plays the most important role in the network. In addition, these three kinds of stakeholders come from three levels: the diffusion source level, the promotion channel level and the adopter level, which means that agriculture green technology diffusion involves various stakeholders and that stakeholders at each level play a part in this process.

Furthermore, the degree centrality, betweenness centrality and closeness centrality of F10 (high cost of green technology diffusion) and F11 (limited market demand for green technology) are the top two factors in the centrality analysis of barriers. This signifies that these two barriers are key difficulties in agriculture green technology diffusion and should be paid more attention to in future research and development. In addition, other barriers, such as F4 (non-significant benefits of green technology), F6 (limited knowledge of farmer), F7 (unscientific green technology promotion) and F12 (low level of rural economy development), are among the top barriers, which indicates that more stakeholders are needed to address these barriers together.

#### 4.2.2. Core–Periphery Network Structure of the Stakeholder–Barrier Network

The core–periphery analysis result of the stakeholder–barrier network is displayed in a density matrix (Table 3), and the final fitness is 0.624. The interactive density between the stakeholders and barriers is 0.778, which shows that stakeholders and barriers at the core are closely related. The density between core stakeholders and peripheral barriers and that between the core barriers and peripheral stakeholders are 0.545 and 0.593, respectively, which are significantly lower than the density index of core sub-divisions. This indicates a loose relationship between core stakeholders and peripheral barriers and a distant relationship between core obstacles and peripheral stakeholders. Therefore, the stakeholder–barrier network exhibits a core–periphery structure.

The core stakeholders and barriers (Table 4) are determined: there are six stakeholders and eight barriers at the core. The six stakeholders are S1 (Agricultural Research Institute), S2 (university), S3 (agribusiness), S10 (government), S6 (agency of township promotion) and S14 (farmers’ relatives). The eight barriers at the core account for two-thirds of the total, with the exclusion of F2 (poor green technology applicability), F3 (poor green technology operability), F5 (non-significant benefits of green technology) and F9 (lack of codes and standards of green technology). Since core stakeholders are more capable of tackling these barriers, intensive interactions between core stakeholders are likely to occur. This boosts information exchange between core stakeholders, who may contribute to the formation of common values, attitudes and benefits in agriculture green technology.

The core–periphery structure in this study can provide guidance for establishing an active collaboration network for stakeholders. For instance, S10 (government), located in the core, is able to solve 11 barriers, among which eight are in the core and four are in the periphery. As shown in Table 4, the eight core barriers may also be addressed by other core stakeholders. As a result, a collaboration between the core stakeholders and S10 (government) should be established. Given its high centrality degree, S10 (government) should play a key mediating role in the partnership between these core stakeholders. According to Table 4, the eight peripheral stakeholders listed may be able to deal with these core barriers even if they have a weaker influence than core stakeholders. Therefore, core stakeholders can team up with peripheral counterparts to solve core barriers. The four peripheral barriers can be solved by both core and peripheral stakeholders. For instance, F11 (limited market demand of green technology) can be affected and addressed by the peripheral stakeholder S8 (Seller), as well as by the four core stakeholders, namely, S10 (government), S3 (agribusiness), S6 (agency of township promotion) and S14 (farmers’ relatives).

### 4.3. Stakeholder/Barrier Network Analysis

#### 4.3.1. Stakeholder/Barrier Network Measures

It is indispensable to transform the two-mode stakeholder–barrier network into two one-mode networks of barrier–barrier and stakeholder–stakeholder. The density and cohesion of the one-mode networks of barrier–barrier and stakeholder–stakeholder are analyzed to understand the characteristics of the whole networks.

The density of the barrier network and stakeholder network is 0.999 and 0.967, respectively, indicating that they have relatively high density and that the relationships between barriers and stakeholders are complicated. The cohesion index of the barrier network is 0.985, signifying a close and complicated relationship among barriers. The cohesion index of the stakeholder network is 0.973, showing that stakeholders can contact each other. When a barrier emerges, stakeholders participating in the program of agriculture green technology may all be involved. Statistics of the cohesion index show that the relationship among stakeholders is close and intricate.

#### 4.3.2. Analysis of the in/out-Degree of the Stakeholder/Barrier Network

**Figure 2:**
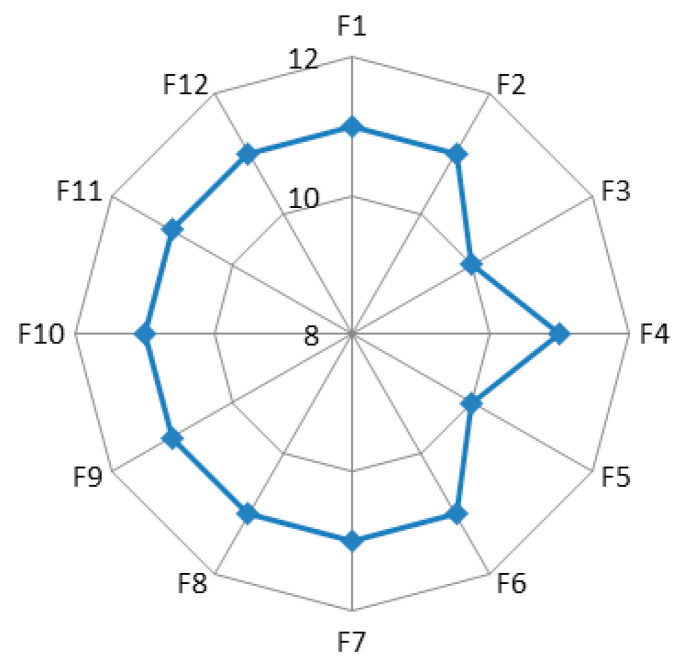
In/out-degree for the one-mode barrier social network.

As a result of the symmetrical nature of the barrier network, the indegree of each node equals the outdegree. In addition, the standard deviation is 0.373, indicating that the influence of each barrier is relatively concentrated. Figure 2 shows the degree of influence of each barrier. As shown in the figure, the highest degree of the barriers below is 11, including F1 (high cost of green technology innovation), F2 (poor green technology applicability), F4 (non-significant benefits of green technology), F6 (limited knowledge of farmer), F7 (unscientific green technology promotion), F8 (inadequate implementation of policies), F9 (lack of codes and standards of green technology), F10 (high cost of green technology diffusion), F11 (limited market demand of green technology) and F12 (low level of rural economy development). This reveals that they are more likely to lead to the occurrence of other barriers. By contrast, the degree of influence of F3 (poor green technology operability) and F5 (farmer families in distress) is 10, which is lower than the average level of 11, showing that they will cause a less direct impact on other barriers.

Similar to the barrier network, the in/out-degree of each node of the stakeholder network remains the same. The overall degree is 176, while the average degree of the stakeholders is 12.57. Thus, a given stakeholder will influence an average of 12.57 stakeholders, including the original stakeholder, and, in turn, these stakeholders will also influence the original stakeholder. The influence of a particular stakeholder on others is relatively concentrated and vice versa. The standard deviation is 0.821.

**Figure 3:**
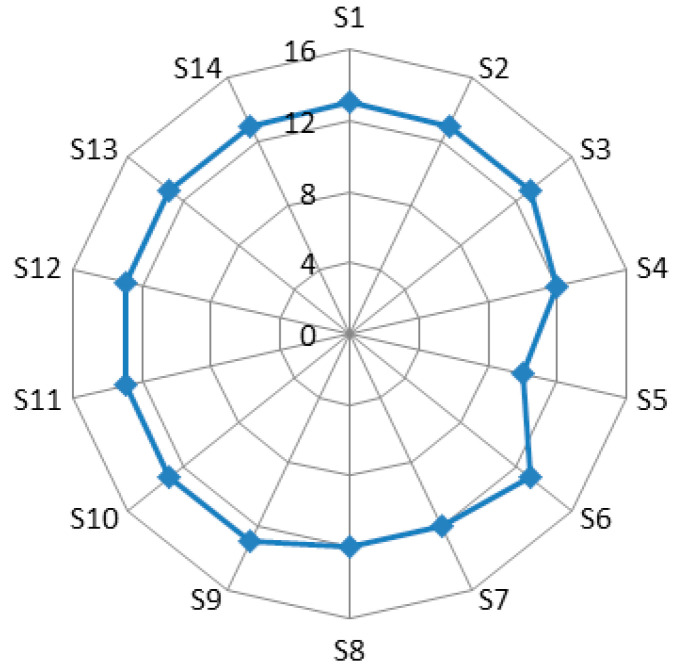
In/out-degree for the one-mode stakeholder social network.

Figure 3 shows the in/out-degree of stakeholders. S1 (Agricultural Research Institute), S2 (university), S3 (agribusiness), S6 (agency of township promotion), S9 (technical promoter), S10 (government) S11 (intermediary institutions), S12 (farmer), S13 (competitor) and S14 (farmers’ relative/relatives of farmer) all reach the highest level of 13 (higher than the average of 12.57). Therefore, these stakeholders will have the greatest impact on barriers and play an important part in the stakeholder network. In contrast, S8 (seller), S4 (supplier), S7 (media) and S5 (researcher) have less impact on other stakeholders; that is, they have little impact on addressing barriers. Among them, sellers, suppliers and media, of which the in/out-degree is 12, play a more important role than scientific researchers, of which the in/out-degree is 10, in addressing barriers.

#### 4.3.3. Structural Hole Analysis of the Stakeholder/Barrier Network

The measurement of a structural hole will help identify barriers that have a great impact on other non-associated barriers in the barrier–barrier network, as well as stakeholders that have a stronger influence on other non-associated stakeholders in the stakeholder–stakeholder network.

**Figure 4:**
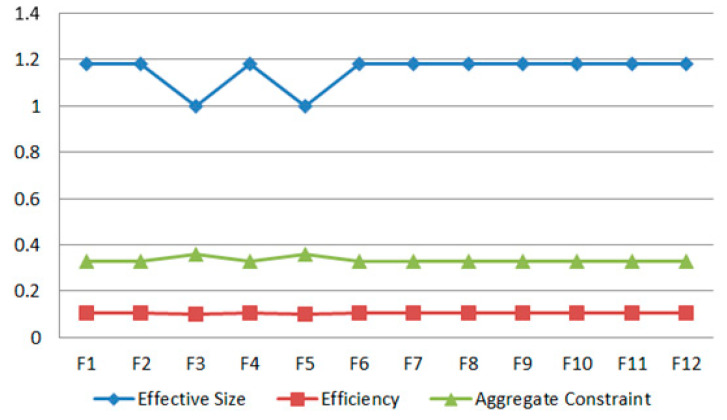
Structural holes for the barrier network.

Figure 4 shows the measurement results of the structural holes in the barrier–barrier network. The values of Y-axis are the results for measuring the structural hole by effective size, efficiency and aggregate constraint. It is clear that the efficiency of the structural holes in the barrier network is consistent with the distribution of effective size, while it has a negative correlation with the aggregate constraint. F3 (poor green technology operability) and F5 (farmer families in distress) are among the weaker barriers in the barrier network. Their small effective size and efficiency indicate a relatively low level of controllability. However, the aggregate constraint of these two barriers is relatively high, which means that they play a weaker role in the barrier network, as they are constrained by the other ten barriers.

**Figure 5:**
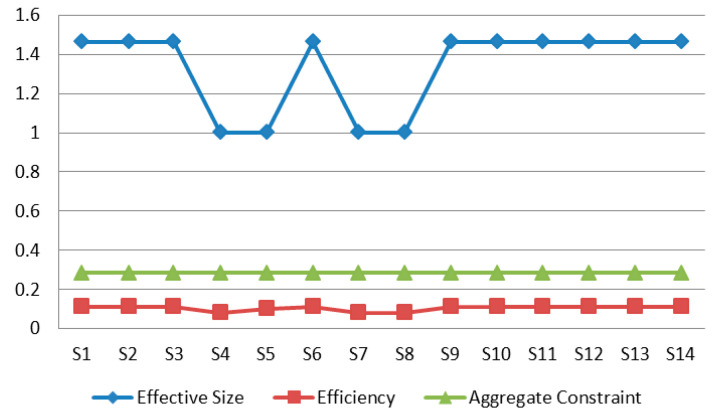
Structural holes for the stakeholder network.

The measurement results of the structural holes in the stakeholder network are shown in Figure 5. To be specific, the effective size of S4 (supplier), S5 (researcher), S7 (media) and S8 (seller) is relatively small, while the efficiency is relatively low. This demonstrates weak controllability. In addition, they have a strong aggregate constraint, showing that they do not play a key role in the control of barriers.

## 5. Discussion

Agriculture is being upgraded with the advancement of the era. Green agriculture has gained unprecedented attention in China. Quite a few scholars have conducted research into agriculture green technology diffusion from different perspectives. However, most of them only analyzed barriers or stakeholders and put forward advice in this regard. Only a few of them have focused on the key subject of stakeholders overcoming barriers. This study offers new perspectives for exploring collaboration among stakeholders in agriculture green technology diffusion. The results of this analysis are discussed in this section.

The core and most important stakeholders identified in the two-mode network analysis are agricultural research institutes, universities, agribusiness, the government, agencies of township promotion and farmers’ relatives, which can influence other stakeholders with their power and reputation, and they are more likely to address core barriers. The key barriers are the limited market demand for green technology and the high cost of its diffusion, which are also found in the one-mode network analysis. Furthermore, the results of the one-mode network analysis also show that poor green technology operability and farmer families in distress are factors that are not as important as previously perceived, and the relatives of farmers and township promotion agencies are becoming very important in social networks. From the analysis of both the two-mode and one-mode networks, it is apparent that the government has the strongest power over the barriers and can affect other stakeholders. Therefore, it is important for the government to use its power to establish collaboration among the stakeholders, especially those who are in the core position and can best address the barriers.

(1) Survey results and findings show that “high cost of green technology diffusion” and “limited market demand for green technology” are deemed important barriers in the social network. Although agriculture green technology has gained increasing attention from all walks of society, it is still developing slowly. The majority of stakeholders are accustomed to traditional agriculture technologies. Many of them, such as agribusinesses and farmers, hold a negative attitude toward adopting agriculture green technology. They pay considerable attention to the cost of agriculture green technology diffusion during the research, innovation, production, promotion and application process [45], which causes the cost of agriculture green technology adoption to become a key factor of whether they will adopt agriculture green technology or not. In remote rural areas and ethnic minority-inhabited regions in West China in particular, the increase in diffusion costs has directly affected the adoption of agriculture green technology. Therefore, high diffusion costs will not only lead to negative attitudes of most stakeholders toward agriculture green technology, but also increase the proportion of farmers who adopt traditional agriculture technology. These two factors may significantly reduce the market demand for agriculture green technology, thus reducing its supply [1]. This could also explain why the “high cost of green technology diffusion” and “limited market demand for green technology” are regarded as major barriers to the diffusion of green technologies during the promotion and diffusion process.
(2) As a key stakeholder in the agriculture green technology diffusion in China, the “government” has a clear central position in the network and has a strong influence. It can influence more barriers than other stakeholders and plays an important role in promoting stakeholder collaboration. Although the Chinese government has attached great importance to the development and diffusion of agriculture green technology in recent years, it has not diffused as well as expected. As a result of externalities and the path dependence effect [49], the innovation and diffusion of agriculture green technology require policy support from the government to reach an optimal level. Effective government environmental policy is a necessary intervention that will exert pressure on enterprises to reduce emissions [50], form shadow prices of emissions and help induce the innovation and diffusion of green technology by many stakeholders. As the main advocate of agriculture green technology, the government’s incentives, supervision and management have a direct impact on its diffusion and promotion. However, as a result of imperfect laws, regulations and supervision systems, government power is limited, and cannot reach all areas in many sparsely populated rural areas, resulting in the failure to implement agriculture green technology [24].
(3) Two barriers—poor green technology operability and farmer families in distress—are not as important as previously perceived, which can be seen from the results of social network analysis. In recent years, China has attached great importance to the development of green agriculture [4], and the trading market for agriculture green technology has gradually matured. The strength of research and development, maturity, usefulness and ease of use of agriculture green technology have also been reinforced by rigorous market testing and screening [51]. For farmers with a low level of cultural knowledge, its operability can still be effective, so it is less important than public awareness. Second, in order to achieve the “two centenary goals”, China has made two strategic decisions—rural revitalization and poverty alleviation—at the national level, greatly improving the living standards of farmers and gradually realizing common prosperity by eliminating poverty and improving people’s livelihood. Therefore, the barrier “farmer families in distress” is becoming less important, and is no longer the main barrier to the diffusion of agriculture green technology.
(4) As expected, six core stakeholders, namely, agricultural research institutes, universities, agribusiness, government, agencies of township promotion and farmers’ relatives, can influence other stakeholders with their power and reputation, and they are more likely to address core barriers. Whether the core stakeholders can achieve effective cooperation and collaboration has greatly affected the development of agriculture green technology. The entire process of the diffusion of agriculture green technology, from the establishment of goals to the realization of technology and the determination of the final operational flow, requires effective coordination among multiple stakeholders. The information exchange, intensive interaction and cooperation among the core stakeholders, including agricultural research institutes, universities, agribusiness, government, agencies of township promotion and farmers’ relatives, will facilitate common values, attitudes and interests in agriculture green technology, thus improving its acceptance by other stakeholders and further promote its diffusion [52]. Furthermore, given its high centrality degree, the government should play a key intermediary role in promoting partnerships among these core stakeholders. Rural areas need the government to design better rules and regulations to greatly enhance the level of agriculture green technology, because of its system, scale and industrial characteristics.
(5) An interesting finding is that relatives of farmers and township promotion agencies are becoming very important in social networks. At present, farmers’ relatives and township promotion agencies need to further deepen their understanding of agriculture green technology. Although an increasing number of adopters and farmers realize that ecological and environmental damage is a serious problem, they often consider it to be an issue that needs to be addressed by the government, and few farmers are concerned about the adoption of agriculture green technology. As a result of the structure of rural society, a relatively fixed social network is established in relatively closed spaces, social norms are relatively easy to form, and the social relationship between individuals is more affected by factors such as blood kinship and geographical scope, which can significantly affect the adoption of green technology by farmers [53,54]. At the same time, because of dispersed settlement, decentralized management and a low degree of organization in rural areas, the diffusion of agriculture green technology is hindered. Therefore, farmers’ relatives and township promotion agencies play an increasingly important role in the diffusion and promotion of agriculture green technology.

## 6. Conclusions

Agriculture green technology is considered to be a useful tool to address various development issues, but it is still in its infancy in many countries, especially in developing countries like China. In the process of studying agriculture green technology diffusion, stakeholder collaboration as a key factor has been neglected by scholars, and it is not clear who among the stakeholders can solve which barrier. Through the analysis of the stakeholder–barrier network, this paper links the identified barriers to stakeholders and provides an innovative perspective for exploring stakeholder collaboration in the process of agriculture green technology diffusion.

With the results of the social network analysis method, this paper discusses the influence of stakeholders on various barriers to agriculture green technology diffusion. Two-mode social networks, one-mode stakeholder networks and one-mode barrier networks are all used to study the relationship between stakeholders and barriers. This expands the traditional research model on stakeholders and barriers and provides theoretical and practical significance for promoting the diffusion of agriculture green technology. The following important findings are drawn from this study: (1) Compared with other barriers, “high cost of green technology diffusion” and “limited market demand of green technology” have more serious impacts on the diffusion of agriculture green technology. (2) The government, as the most influential stakeholder, plays an important role in overcoming barriers to the diffusion of agriculture green technology and promoting stakeholder collaboration. (3) Some barriers, such as poor green technology operability and farmer families in distress, are not as important as usually perceived. (4) Collaboration among core stakeholders, such as agricultural research institutes, universities, agribusiness, the government, agencies of township promotion and farmers’ relatives, can influence other stakeholders and facilitate the resolution of barriers. Effective promotion of collaboration among core stakeholders plays an extremely important role in overcoming barriers to the diffusion of agriculture green technology. (5) Farmers’ relatives and agencies of township promotion are becoming very important in social networks. Although these research results need to be tested with more in-depth practical investigations and academic research, the results of this study can provide stakeholders with new ideas to remove barriers to the diffusion of agriculture green technology, and can serve as a policy- and decision-making reference for government management departments and enterprises to facilitate this diffusion.

On the basis of the results of the analysis, this paper proposes some suggestions for promoting the diffusion of agriculture green technology. First, in order to successfully diffuse and widely implement agriculture green technologies in China, it is necessary to further actively respond to and implement the two strategic decisions of rural revitalization and poverty alleviation, continuously improve the living standards of farmers’ families, promote the development of green agriculture as a whole, boost the performance of agriculture green technology diffusion, reduce the cost of diffusion and vigorously tap the green technology market, thereby increasing the demand for agriculture green technology.

Second, as the main advocate of agriculture green technology, the government’s environmental policies can help encourage many stakeholders to cooperate in the diffusion of green technology innovation, thus addressing many diffusion barriers. If environmental policy tools, such as carbon tax and subsidies, can be integrated and synergistically combined, then the results will be better.

Third, the government should actively respond to the trend of agricultural development and promote collaboration among core stakeholders, such as agricultural research institutes, universities, agribusiness, government, agencies of township promotion and farmers’ relatives through policy incentives and regulation design, and it should give full play to the basic role of market mechanisms in resource allocation, build an innovation and diffusion system of agriculture green technology based on stakeholder coordination, promote the supply of green technology innovation, reduce the cost, improve efficiency and further promote the diffusion of agriculture green technology.

Finally, it is necessary to attach importance to the role of farmers’ relatives and agencies of township promotion in the diffusion of agriculture green technology, raise farmers’ awareness of green development, promote awareness of agriculture green technology among farmers’ relatives and township promotion agencies and strengthen their communication to promote barrier removal with policy guidance and social education. Farmers’ relatives and township promotion agencies should be encouraged to actively participate in the diffusion of agriculture green technology with policy incentives and benefit-sharing mechanisms.

This study also has some limitations. First, one limitation of this study is that the data are from China, where agriculture green technology is in its initial stage of development, so the results of this study can only reflect the role of stakeholders in the diffusion of agricultural green technology in China, and not in countries in which the development of agriculture green technology is relatively mature.

For future research, international comparison research can be conducted in these countries in order to establish international benchmarks for the acceptance of agriculture green technology. As the diffusion of agricultural green technology is a complex process, involving basic research, applied research and on-farm research to adapt new techniques to local conditions, participatory extension strategies, the establishment of farmer-to-farmer networks and the acquisition of an in-depth understanding of household dynamics, decision-making and community socioeconomic conditions, a modeling approach can be considered the first step to assess the dynamics of technology transfer and adoption. Future research may place a focus on carrying out follow-up on-farm research and surveys to validate, strengthen or fine-tune the findings from a valuable modeling approach, so as to better understand interactions among stakeholders and barriers to diffusion. Additionally, agriculture green technologies may be better adopted among some groups of farmers compared with those with less education or more limited resources. Future research can be carried out on target farm groups with similar socioeconomic backgrounds, to clarify the relationship between stakeholders and diffusion barriers and identify crucial factors that affect adoption and diffusion among groups of farmers with different socioeconomic backgrounds.

## Figures and Tables

**Table 1:** Barriers and stakeholders affecting agriculture green technology diffusion.

Code	Barriers	Code	Stakeholders
F1	High cost of green technology innovation	S1	Agricultural Research Institute
F2	Poor green technology applicability	S2	University
F3	Poor green technology operability	S3	Agribusiness
F4	Non-significant benefits of green technology	S4	Supplier
F5	Farmer families in distress	S5	Researcher
F6	Limited knowledge of farmer	S6	Agency of township promotion
F7	Unscientific green technology promotion	S7	Media
F8	Inadequate implementation of policies	S8	Seller
F9	Lack of codes and standards of green technology	S9	Technical promoter
F10	High cost of green technology diffusion	S10	Government
F11	Limited market demand for green technology	S11	Intermediary institutions
F12	Low level of rural economy development	S12	Farmer
		S13	Competitor
		S14	Farmers’ relatives

**Table 2:** The centrality of nodes in the stakeholder–barrier network.

Stakeholders	DC	Rank	CC	Rank	BC	Rank	Barriers	DC	Rank	CC	Rank	BC	Rank
S1	0.667	3	0.826	3	0.047	4	F1	0.571	7	0.750	7	0.046	7
S2	0.667	3	0.826	3	0.047	4	F2	0.357	10	0.667	10	0.013	11
S3	0.750	2	0.864	2	0.069	2	F3	0.357	10	0.643	11	0.014	10
S4	0.333	14	0.679	13	0.006	14	F4	0.714	3	0.818	3	0.077	3
S5	0.417	11	0.655	14	0.014	11	F5	0.214	12	0.600	12	0.003	12
S6	0.667	3	0.826	3	0.042	6	F6	0.643	5	0.783	5	0.064	5
S7	0.417	11	0.704	11	0.009	13	F7	0.643	5	0.783	5	0.052	6
S8	0.417	11	0.704	11	0.010	12	F8	0.571	7	0.750	7	0.037	8
S9	0.500	7	0.760	7	0.018	9	F9	0.429	9	0.692	9	0.020	9
S10	0.917	1	0.950	1	0.116	1	F10	0.786	1	0.857	1	0.090	1
S11	0.500	7	0.760	7	0.017	10	F11	0.786	1	0.857	1	0.089	2
S12	0.500	7	0.760	7	0.027	8	F12	0.714	3	0.818	3	0.076	4
S13	0.500	7	0.760	7	0.030	7							
S14	0.667	3	0.826	3	0.048	3							

DC = degree centrality, CC = closeness centrality, BC = betweenness centrality.

**Table 3:** Density matrix.

		Barrier	
		Core	Periphery
Stakeholder	Core	0.778	0.545
	Periphery	0.593	0.167
	Overall network density: 0.526		
	Final fitness: 0.624		

**Table 4:** Core–periphery structure model of the stakeholder–barrier network.

	F1	F8	F6	F4	F11	F12	F7	F10	F2	F3	F5	F9
S1	1		1	1		1		1	1	1		1
S2	1		1	1		1		1	1	1		1
S3	1			1	1	1	1	1	1	1		1
S14		1	1	1	1	1	1	1			1	
S6	1	1	1	1	1		1	1				1
S10	1	1	1	1	1	1	1	1	1		1	1
S5			1	1					1	1		1
S8	1				1	1	1	1				
S9		1	1	1	1		1	1				
S4	1				1		1	1				
S11		1			1	1	1	1				
S12		1	1	1	1	1					1	
S13	1	1	1	1	1	1				1		
S7		1			1	1	1	1				
